# Hierarchical modelling of blood lipids’ profile and 10-year (2002–2012) all cause mortality and incidence of cardiovascular disease: the ATTICA study

**DOI:** 10.1186/s12944-015-0101-7

**Published:** 2015-09-15

**Authors:** Tzortzis Nomikos, Demosthenes Panagiotakos, Ekavi Georgousopoulou, Vassiliki Metaxa, Christina Chrysohoou, Ioannis Skoumas, Smaragdi Antonopoulou, Dimitrios Tousoulis, Christodoulos Stefanadis, Christos Pitsavos, and the ATTICA Study group

**Affiliations:** Department of Nutrition and Dietetics, School of Health Science and Education, Harokopio University, Athens, Greece; First Cardiology Clinic, School of Medicine, University of Athens, Athens, Greece; 46 Paleon Polemiston St., Glyfada, Attica 166 74 Greece

**Keywords:** Cardiovascular disease, All cause mortality, Lipids, Lipoproteins, Apolipoproteins, Epidemiology

## Abstract

**Background:**

The traditional view on the relationship between lipid biomarkers and CVD risk has changed during the last decade. However, it is not clear whether novel lipid biomarkers are able to confer a better predictability of CVD risk, compared to traditional ones.Under this perspective, the aim of the present work was to evaluate the predictive ability of blood lipids’ profile on all cause mortality as well as 10-year incidence of CVD, in a sample of apparently healthy adults of the ATTICA epidemiological study.

**Methods:**

From May 2001 to December 2002, 1514 men and 1528 women (>18 y) without any clinical evidence of any other chronic disease, at baseline, were enrolled. In 2011–12, the 10-year follow-up was performed in 2583 participants (85 % follow-up participation rate). Incidence of fatal or non-fatal CVD was defined according to WHO-ICD-10 criteria. Baseline serum blood lipids’ profile (Total-C, HDL-, non HDL-, LDL-cholesterol, triglycerides (TG), apolipoprotein (Apo)A1 and B, and lipoprotein–(a) levels were also measured.

**Results:**

The 10-year all-cause mortality rate was 5.7 % for men and 2.0 % for women (*p* = 0.55). The, 10-year CVD incidence was 19.7 % in men and 11.7 % in women (*p* < 0.001). Multi-adjusted analysis revealed that TC, non-HDL-C, TG and TG/HDL-C ratio, were independent predictors of all cause mortality (RR per 1 mg/dL or unit (95 % CI): 1.006 (1.000–1.013), 1.006 (1.000–1.013), 1.002 (1.000–1.004), 1.038 (1.001–1.077), respectively). Moreover, TC, HDL-, LDL-, non-HDL-cholesterol, TG, apoA1, TC/HDL-C and TG/HDL-C were independently associated with CVD risk. Among all lipid indices the ratio of apoB/apoA1 demonstrated the best correct reclassification ability, followed by non-HDL-C and TC/HDL-C ratio (continuous Net Reclassification Index 26.1 and 21.2 %, respectively).

**Conclusion:**

Elevated levels of lipid biomarkers are independently associated with all-cause mortality, as well as CVD risk. The ratio of apoB/apoA1, followed by non-HDL-C, demonstrated the best correct classification ability of the developed CVD risk models.

## Background

Cardiovascular disease (CVD) remains one of the major causes of death world-wide, despite the huge efforts that have been undertaken the past decades for the clarification of its pathogenesis and treatment, as well as its prevention at population and individual level [1]. The identification of the risk factors that lead to atherosclerosis and their subsequent modification by lifestyle interventions and pharmaceutical treatment is the cornerstone of the prevention policies [2]. Large cohort studies have shown that smoking and dyslipidemia are the two most important risk factors for myocardial infarction, followed by diabetes, hypertension and obesity [3]; and most scoring systems utilize age, gender, systolic blood pressure, smoking status, and total cholesterol (TC) or Low Density Lipoprotein cholesterol (LDL-C) concentration as the main variables of their algorithms [4–6]. Nowadays, the reduction of LDL cholesterol levels remains the primary target for the primary prevention of CVD [7, 8] and this is supported by a strong body of evidence showing that it is an important marker of coronary heart disease (CHD) [9]. However, it has been acknowledged that a high residual CVD risk still exists, with a considerable proportion of potential CVD “candidates” being underestimated, and therefore, the need for the identification of novel biomarkers, especially of lipid/lipoprotein metabolism, is emerged [10]. Moreover, a high residual risk characterizes people with obesity or metabolic syndrome, where LDL-C is less predictive for developing CVD [11]. These people are characterized by low levels of HDL-C, elevated levels of triglycerides and a high content of small dense pro-atherogenic apo B lipoprotein particles [12]. Small dense lipoproteins (which is estimated by the apoB or non-HDL cholesterol concentration), penetrate more easily the arterial wall and it seems that their number rather than their cholesterol content drives foam cell formation [13]. It is therefore possible that people with a high content of elevated small dense lipoprotein particles have near normal LDL-C values due to the discordance between the apoB particle number and their cholesterol content. These people will have an underestimated risk prediction score [14]. Moreover, accumulating evidence from recent epidemiological, genetic and biochemical studies changed the traditional view for the role of HDL-C which seems to serve as a strong predictor rather than a causative factor of CVDs [15, 16]. Finally, recent cohort studies have demonstrated the predictive power of Lp(a) since its elevated levels correlate to CVD risk in a continuous and independent manner [17]. It is therefore obvious that the traditional view on the relationship between lipid biomarkers and CVD risk has changed during the last decade. However, it is not clear whether novel lipid biomarkers (e.g., apoB, apoA1, Lp(a), non-HDL-C) are able to confer a better predictability of CVD risk, compared to the more traditional ones (LDL-C, HDL-C and TGs).

Based on the existing literature, studies evaluating the predictive ability of a variety of blood lipids/lipoproteins for CVD incidence are lacking. Under this perspective, the aim of the present work was to evaluate the predictive ability of blood lipids’ profile, i.e., TC, LDL-C, HDL-C non-HDL-C, apoA1, apoB and Lp(a) levels, on all cause mortality as well as 10-year incidence of CVD, in a sample of apparently healthy adults of the ATTICA epidemiological study.

## Methods

### Sampling procedure at baseline examination

The ATTICA study was established during 2001–2002, in the greater metropolitan area of Athens (that includes 78 % urban and 22 % rural regions). The sampling procedure anticipated enrolling only one participant per household; it was random, multistage and based on the age, sex distribution of the region (as provided in the 2001census). Of the 4056 initially invited individuals, 3042 agreed to participate at baseline examination (75 % participation rate); 1514 of the participants were men (18–87 years) and 1528 were women (18–89 years). All participants were interviewed by trained personnel (cardiologists, general practitioners, dieticians and nurses) who used a standard questionnaire. Exclusion of CVD at baseline evaluation was performed through a detailed clinical evaluation by the physicians of the study, following standard criteria. The examination was performed in the individuals’ homes or workplaces places.

### Lipids and other measurements at baseline examination

The blood samples were collected from the antecubital vein, between 8 to 10 a.m., in a sitting position after 12 h of fasting and avoiding of alcohol. Serum for the measurement of blood lipids was harvested immediately after admission. The biochemical evaluation was carried out in the same laboratory that followed the criteria of the World Health Organization Lipid Reference Laboratories. Serum total cholesterol, HDL-cholesterol and triglycerides were measured using chromatographic enzymic method in a Technicon automatic analyser RA-1000 (Dade Behring, Marburg, Germany). HDL-cholesterol was determined after precipitation of the Apolipoprotein B containing lipoproteins with dextran-magnesium-chloride. Non-HDL cholesterol was calculated by the formula: total cholesterol minus HDL cholesterol. Lipoprotein (a) was measured by a latex enhanced turbidimetric immuno-assay. LDL cholesterol calculated using the Friedewald formulae: {total cholesterol} – {HDL cholesterol} – 1/5 (triglycerides) (only for participants with triglycerides < 400 mg/dL). ApoB and apoAI were measured by rate immunonephelometry. An internal quality control was in place for assessing the validity of cholesterol, triglyceride and HDL methods. The intra and inter-assay coefficients of variation of cholesterol levels did not exceed 9 %, triglycerides 4 % and HDL 4 %.

The baseline evaluation also included information about: age, sex, mean annual income and years of school, assessment of history of hypertension, hypercholesterolemia and diabetes, family history of CVD, dietary and lifestyle habits (i.e., smoking status, dietary habits and physical activity status). Specifically, smokers were defined those who were smoking at least one cigarette per day during the past year or had recently stopped smoking (during a year), while the rest of the participants were defined as noncurrent-smokers; the evaluation of the dietary habits was based on a validated semi-quantitative food-frequency questionnaire [18], the EPIC-Greek questionnaire, which was kindly provided by the Unit of Nutrition of Athens Medical School. The MedDietScore was also applied (range 0–55) to evaluate adherence to the Mediterranean diet [19]. For the evaluation of physical activity status the International Physical Activity Questionnaire (IPAQ) was used [20], as an index of weekly energy expenditure using frequency (times per week), duration (in minutes per time) and intensity of sports or other habits related to physical activity (in expended calories per time). Participants who did not report any physical activities were defined as physically inactive (sedentary lifestyle). Body mass index (BMI) was measured as weight (in kilograms) divided by standing height (in meters squared). Obesity was defined as BMI greater than 29.9 Kg/m^2^. Waist (in cm) and hip (in cm) circumferences were also measured. Arterial blood pressure (3 recordings) was measured at the end of the baseline physical examination with subject in sitting position, and at least 30 min at rest. Participants whose average blood pressure levels were greater or equal to 140/90 mmHg or were under antihypertensive medication were classified as having hypertension. Further details about the aims and procedures of the ATTICA epidemiological study may be found elsewhere [21].

### Follow-up examination (2011–2012)

During 2011–12, the ATTICA Study’s investigators performed the 10-year follow-up (mean follow-up time 8.41 y). Of the *n* = 3042 initially enrolled participants, *n* = 2583 were found during the follow-up (85 % participation rate). Of the *n* = 459 individuals that were lost to follow-up, *n* = 224 were not found because of missing or wrong addresses and telephone numbers that they have provided at baseline examination and *n* = 235 because they denied to be re-examined. In order to participate in the follow-up all participants were initially appointed through telephone calls. Afterwards, the investigators approached the allocated participants and performed a detailed medical evaluation. Information about participants’: (a) vital status (death from any cause), (b) development of fatal or non-fatal CVD (i.e., myocardial infarction, angina pectoris, other identified forms of ischemia -WHO-ICD coding 410–414.9, 427.2, 427.6-, heart failure of different types and chronic arrhythmias -WHO-ICD coding 400.0–404.9, 427.0–427.5, 427.9-, stroke WHO-ICD coding 430–438), was also assessed. Accurate CVD incidence data were obtained for *n* = 2,020 out of the 2,583, thus, the analysis for 10-year CVD incidence was performed for these subjects. Further details about the follow-up of the ATTICA epidemiological study may be found elsewhere [22, 23].

### Statistical analysis

All cause mortality rates or non-fatal and fatal CVD incidence was calculated as the ratio of new deaths or CVD cases, respectively, to the number of people participated in the follow-up. Continuous variables were presented as mean values ± standard deviation and categorical variables are presented as frequencies. Associations between categorical variables were tested using the Pearson’s chi-square test. The Kruskal-Wallis and the McNemar non-parametric tests were used to compare continuous factors and prevalence rates of clinical conditions between baseline and follow-up examinations. Comparisons between mean values of normally distributed variables between those who developed an event and the rest of the participants were performed using Student’s *t*-test, after controlling for equality of variances using the Levene’s test. The continuous variables were tested for normality through P-P plots. For the continuous variables that were not normally distributed (i.e., triglycerides) the Mann–Whitney test was applied to evaluate the differences in the distributions between those who developed an event and the rest of the participants. The relative risks (RR) of developing an event during the 10-year period, according to the participants’ baseline characteristics were estimated using Cox proportional hazards models. Proportionality of hazards was graphically assessed by plotting partial residuals against time. The time to CVD event was recorded on annual basis. Interactions between age, sex and lipids levels were tested and *Log*-rank test was applied to evaluate difference between genders. Known confounders (i.e., smoking, physical activity status, BMI, history and management of hypertension, diabetes, hypercholesterolemia and family history of CVD) were also included in all models. Negelkerke adjusted R-square and C-statistic were calculated to evaluate model’s goodness of fit and classification ability. Harrell’s C of inverse RR was used as a measure of the predictive power of the estimated models [24]; Harrell’s C is defined as the proportion of all subject pairs in which the predictions and outcomes are concordant. Harrell’s C plays an important role in rank statistics and has been extensively used for assessing prediction performance in survival analysis settings. The continuous-Net Reclassification Improvement (cNRI) was also calculated, according to Pencina et al., in order to quantify the % of improvement in model’s predictive ability after the introduction of various blood lipids [25]. All reported *p*-values are based on two-sided tests. SPSS version 19 (Statistical Package for Social Sciences, SPSS Inc, Chicago, IL, USA) and STATA version 12 (College Station, TX, USA) software were used for all the statistical calculations.

## Results

The 10-year all-cause mortality rate was 3.8 % (*n* = 73, 5.7 % of men and *n* = 26, 2.0 % of women had a fatal event, *p* = 0.55). The distribution of causes of deaths was: 51.1 % were due to CVD (i.e., 42.2 % CHD, 4.4 % stroke and 4.4 % other CVD), 30.0 % were due to cancer, 7.8 % were due to infections (mainly pulmonary), 6.7 % were accidents and the rest 4.4 % were due to other reasons not identified. The, overall 10-year CVD incidence was *n* = 317 (15.7 %) (*n* = 198, 19.7 % in men and *n* = 119, 11.7 %in women, *p* < 0.001). Of the *n* = 317 CVD events, 46 were fatal (*n* = 34 men); the 10-year CVD death rate was 1.8 % (3.4 % for men and 1.2 % for women, *p* < 0.001).

Blood lipids profile, demographic, clinical and lifestyle characteristics of the participants according to the 10-year event status are presented in Table 1. Participants who developed a CVD event within the 10-year follow-up were older at baseline examination than subjects who remained free of the disease (*p* < 0.001), more likely to be men (*p* < 0.001), with lower educational status (*p* < 0.001), obese (*p* < 0.001) and they were away from the Mediterranean diet, as assessed with the MedDietScore (23 ± 7/55 vs. 26 ± 6/55, respectively, *p* < 0.001). As regards their baseline clinical profile, subjects who developed CVD were more likely to be hypertensive (*p* < 0.001), to have diabetes mellitus (*p* < 0.001) and hypercholesterolemia (*p* < 0.001), than CVD-free subjects. Blood lipids levels were also elevated among subjects who developed a CVD-event.

**Table 1:** Characteristics of the ATTICA study’s participants (*n* = 2020) according to the 10-year fatal or non-fatal incidence of CVD

		Status at 10-year follow – up		
	Baseline	CVD event free (*n =* 1703)	CVD events (*n* = 317)	*p*
Age, yrs	45 ± 14	43 ± 13	58 ± 13	<0.001
Male gender, %	50 %	48 %	63 %	<0.001
Years of school	12 ± 4	13 ± 4	10 ± 4	<0.001
Smoking at baseline or before, %	43 %	55 %	57 %	0.462
Physical activity, %	41 %	41 %	41 %	0.999
MedDietScore (0–55)	26 ± 7	26 ± 6	23 ± 7	<0.001
Hypertension, %	30 %	28 %	51 %	<0.001
Hypercholesterolemia, %	39 %	40 %	57 %	<0.001
Use of lipid-lowering meds, %	25 %	38 %	21 %	<0.001
Total cholesterol, mmol/L	4.99 ± 1.09	4.99 ± 1.06	5.33 ± 1.09	<0.001
HDL-cholesterol, mmol/L	1.24 ± 0.39	1.27 ± 0.39	1.16 ± 0.31	<0.001
LDL-cholesterol, mmol/L	3.15 ± 0.96	3.13 ± 0.96	3.39 ± 1.03	<0.001
Apolipoprotein AI, mg/dL	154 ± 27	156 ± 27	150 ± 24	0.001
Apolipoprotein B, mg/dL	108 ± 38	106 ± 42	109 ± 29	<0.001
Triglycerides, mmol/L**	1.13 ± 0.42	1.08 ± 0.41	1.48 ± 0.51	<0.001
Lipoprotein-a, mg/dL**	11 ± 9.5	11 ± 9.5	13 ± 11	0.052
Diabetes, %	7 %	5 %	22 %	<0.001
Obesity, %	18 %	17 %	28 %	<0.001
Family history of CVD, %	28 %	28 %	32 %	0.297

*p-values for the comparisons between CVD event and event-free group derived using the *t*-test for age, total, HDL-cholesterol and MedDietScore or Mann–Whitney non-parametric test (**) for years of school and the rest lipids, while for the comparisons of categorical variables using the chi-square test

The association between various lipidaemic markers on CVD risk as well as on all-cause mortality risk was investigated, independently of several potential confounders (age, sex, Body Mass Index, physical activity status, smoking, level of adherence to Mediterranean diet - assessed with the MedDietScore-, history and management of hypertension, dyslipidemia and diabetes mellitus and family history of CVD).

### Modelling blood lipids levels and 10-year all cause mortality

As it can be seen in Table 2 regarding all-cause mortality, it was observed that for 10 units (mg/dL) or 0.26 units (mmol/L) increase in TC levels, the 10-year mortality risk rises by 0.6 % (RR per 1 mg/dL or per 0.026 mmol/L: 1.006, 95 % Confidence Intervals (CI):1.00, 1.013). The same effect was revealed for non-HDL cholesterol (RR per 1 mg/dL or per 0.026 mmol/L: 1.006, 95 %CI: 1.00, 1.013), while for 10 units (mg/dL) or 0.115 units (mmol/L) increase in serum triglycerides, the 10-year mortality risk increased by 0.2 % (RR per 1 mg/dL or per 0.0115 mmol/L: 1.002, 95 % CI: 1.000, 1.004). The only lipidaemic markers ratio of those tested here, that was significantly associated with 10-year all-cause mortality was the triglycerides/HDL-cholesterol ratio, which increased the risk by 3.8 % per 1 unit increase (RR per 1 unit: 1.038, 95 % CI:1.001,1.077).

**Table 2:** Results from survival models that evaluated the association of blood lipids and lipoproteins on 10-year all-cause mortality and CVD incidence, among the ATTICA study participants (*n* = 2,583)

	All-cause mortality		CVD incidence	
Independent predictor	RR	95 % CI	RR	95 % CI
Model 1: Total cholesterol (per 1 mg/dL*)	1.006	1.000,1.013	1.003	0.999,1.007
Model 2: HDL cholesterol (per 1 mg/dL)	0.988	0.962,1.015	0.983	0.967,1.000
Model 3: LDL cholesterol (per 1 mg/dL)	1.004	0.996,1.011	1.002	0.998.1.007
Model 4: non-HDL cholesterol (per 1 mg/dL)	1.006	1.000,1.013	1.005	1.001,1.009
Model 5: Triglycerides (per 1 mg/dL)	1.002	1.000,1.004	1.002	1.001,1.003
Model 5: Lipoprotein-a (per 1 mg/dL)	1.004	0.994,1.015	1.003	0.997,1.010
Model 6: Apolipoprotein-A1 (per 1 mg/dL)	0.991	0.978,1.004	0.992	0.985,1.000
Model 7: Apolipoprotein-B (per 1 mg/dL)	1.004	0.997,1.012	1.002	0.998,1.007
Model 8: Total/HDL-cholesterol ratio (per 1 unit)	1.169	0.978,1.387	1.216	1.092,1.354
Model 9: Apolipoprotein-B/-A1 ratio (per 1 unit)	1.560	0.837,2.910	1.389	0.921,2.094
Model 10: Triglycerides/ HDL-cholesterol ratio (per 1 unit)	1.038	1.001,1.077	1.055	1.031,1.078

All models were adjusted for age, sex, BMI, smoking habits, physical activity status, MedDietScore and history and pharmaceutical management of dyslipidemias, hypertension and diabetes, and family history of CVD at baseline examination. Results are presented as Relative Risk (RR) and 95 % Confidence Interval (CI)

*1 mg/dL corresponds to 0.0259 mmol/L for Total cholesterol, HDL cholesterol, LDL cholesterol, non-HDL cholesterol and to 0.0115 mmol/L for Triglycerides

### Modelling blood lipids levels and 10-year CVD event rate

The association between lipid markers and 10-year CVD incidence was also tested. It was revealed that for 10 units (mg/dL) or 0.26 units (mmol/L) increase in non-HDL-cholesterol levels, the 10-year CVD risk increased by 5 % (RR per 1 mg/dL or per 0.026 mmol/L: 1.005, 95 % CI: 1.001, 1.009), while for 10 units (mg/dL) or 0.115 units (mmol/L) increase in serum triglycerides, the 10-year CVD risk increased by 2 % (RR per 1 mg/dL or per 0.0115 mmol/L: 1.002, 95 % CI:1.001, 1.003). Furthermore, for every unit increase in total cholesterol/HDl-cholesterol ratio, the 10-year CVD risk increased by 21.6 % (RR = 1.216, 95 % CI: 1.092, 1.354) and for every unit increase in triglycerides/HDL-cholesterol ratio, the 10-year CVD risk increased by 5.5 % (RR = 1.055, 95 % CI: 1.031, 1.078).

Of those who had already known blood lipids abnormalities (Table 1), 36 % of men and 33 % of women followed only a dietary medication and 31 % of men and 20 % of women were under a complimentary lipid-lowering medication (95 % of them under statin treatment). The use of lipid-lowering was then taken into account in all models; no alterations in the aforementioned results were observed when a variable indicating whether participants dyslipidemic adhered to the prescribed medication or not entered in the models.

### Evaluating the predictive ability of lipids and lipoproteins on 10-year CVD event rate

One of the main goals of the present analysis was to hierarchy the explanatory ability of lipids levels in predicting future CVD events, and therefore, to provide useful insights in primary CVD prevention. The model with the highest explanatory ability was the one included the total cholesterol/HDL-cholesterol ratio (R-square = 18.5 %), followed by the model with the triglycerides/HDL-cholesterol ratio (R-square = 18.2 %) and the model with the non-HDl-cholesterol (R-square = 18 %), whereas the rest models had an explanatory ability between 17.3-17.9 %. Regarding the predictive power of the estimated models, based on Harrell’s C values it was observed that the best model was the one contained TC/HDL-C levels, followed by the model contained LDL-C and the model contained TGs/HDL-C levels (Tables 3).

**Table 3:** Classification indices from survival models that evaluated the association of blood lipids and lipoproteins on 10-year CVD incidence, among the ATTICA study participants (*n* = 2020)

Independent predictor	Adj. R^2^	Harrell’s C	cNRI
Model 1: Total cholesterol (per 1 mg/dL*)	17.4 %	0.205	10.4 %
Model 2: HDL cholesterol (per 1 mg/dL)	17.8 %	0.521	12.6 %
Model 3: LDL cholesterol (per 1 mg/dL)	17.6 %	0.739	11.1 %
Model 4: non-HDL cholesterol (per 1 mg/dL)	18 %	0.383	21.2 %
Model 5: Triglycerides (per 1 mg/dL)	17.3 %	0.707	5.0 %
Model 5: Lipoprotein-a (per 1 mg/dL)	17.6 %	0.562	6.7 %
Model 6: Apolipoprotein-A1 (per 1 mg/dL)	17.9 %	0.271	3.4 %
Model 7: Apolipoprotein-B (per 1 mg/dL)	17.7 %	0.277	19.7 %
Model 8: Total/HDL-cholesterol ratio (per 1 unit)	18.5 %	0.901	21 %
Model 9: Apolipoprotein-B/-A1 ratio (per 1 unit)	17.7 %	0.417	26.1 %
Model 10: Triglycerides/HDL-cholesterol ratio (per 1 unit)	18.2 %	0.616	14.8 %

Model refers to the ones presented in Table 2

*cNRI* Continuous Net Reclassification Index

*1 mg/dL corresponds to 0.0259 mmol/L for Total cholesterol, HDL cholesterol, LDL cholesterol, non-HDL cholesterol and to 0.0115 mmol/L for Triglycerides

Another important issue in the effective risk management is the accurate prediction of the potential CVD candidate. Identifying factors that better confer to the accuracy of a risk model allows flexibility in risk management and improved assessment of overall risk. Thus, regarding the correct classification rate of the applied CVD risk models, the model containing the Apolipoprotein-B/-A1 ratio was the one with the best correct reclassification ability (continuous Net Reclassification Index (cNRI = 26.1 %), suggesting that addition of this variable in the core model (i.e., the model without the lipid marker, but with all other predictors) lead to improved correct reclassification (CVD event/no-CVD event) of 26 out of 100 participants, followed by the model with the non-HDL-cholesterol (cNRI = 21.2 %). The rest of the models correctly reclassified less than 20 % of the participants to the CVD event or non-event groups (Table 3).

## Discussion

As far as it is known know a comparison of the predictive ability between lipid biomarkers on total mortality, as well as CVD incidence, have rarely been tested before, especially in the Mediterranean region [26, 27]. Taking into account that the ability of each cardiometabolic risk factor to predict a future event could be modulated by lifestyle characteristics [28] and genetic background [29, 30], the evaluation of their predictive ability in various populations is extremely important for the implementation of public health policies. Dyslipidemia is considered as one of the most important risk and prognostic factors for CVD [3, 5, 15, 31]. However, the high residual risk for CVD [10, 11] led lipid research to the suggestion of novel surrogate lipid markers, e.g., apoB, apoA1, non-HDL-C, small dense LDL, LDL particle number, Lp(a) [32, 33], with conflicting results, implying that further studies in different populations are still needed. Moreover, the ability of the aforementioned indices to predict all cause mortality has not been extensively studied. Based on a large scale prospective cohort study, it was revealed here that ApoB/A1 ratio, non-HDL cholesterol levels and TC/HDL-C ratio improved CVD risk models’ predictive ability. Despite the potential limitations of the present study, the results presented here may confer to better understanding the role of lipid biomarkers in CVD risk stratification and management, and therefore, could be taken into consideration by public health care authorities.

Concerning the baseline lipid profile of the study’s participants’, it was characterized by raised levels of TC and a rather high proportion of hypercholesterolemia, but near normal mean values of HDL-C, TGs, Lp(a) and apolipoproteins. Thus, this mild dyslipidemic profile of the cohort along with the relatively small variability of the lipid levels between subjects may modest the strength of the observed association between lipid indices and 10-year CVD risk. Multi-adjusted models demonstrate that lipid indices, specifically TC, non-HDL-C, TGs and the ratio TG/HDL-C, were independent predictors of all cause mortality (Table 2). The positive association between TC and risk of all cause mortality was also observed in a general population cohort from Japan [34], in a middle aged American cohort [35] and in Mexican Americans of the San Antonio Heart Study [36]. Moreover, a recent meta-analysis of prospective studies clearly shows the graded positive association of TG levels and all cause mortality risk [37]. Finally, the ability of TG/HDL-C ratio to be a strong independent predictor of all cause mortality was also observed in acute coronary syndrome patients after coronary revascularization [38] and in women with suspected myocardial ischemia [39]. The positive association between TC, non-HDL-C, TGs, TG/HDL-C and all cause mortality may be partly attributed to their association with CVD, since half of the deaths (51.1 %) were due to cardiovascular deaths. However, a significant proportion of deaths were also due to malignancies (30.0 %) and infections (7.8 %). A worse lipid profile, especially raised TG levels in combination with low HDL-C, is indicative of metabolic syndrome and insulin resistance which in turn associates with common cancers [40] and depression of the immune system [41]. However, since the observed associations were independent of diabetes, other, yet unknown mechanism may underlie this relationship. Whether dyslipidemia is associated with all cause mortality, irrespectively of co-morbidities remains to be elucidated.

Evaluating the ability of lipid biomarkers to predict CVD events it was confirmed that almost all were associated with CVD risk independently from other cardiometabolic risk factors (Table 2). It is therefore obvious that dyslipidemia is an important risk factor for CVD and health policies, aiming initially on lifestyle changes, should be implemented for the improvement of lipids’ metabolism which will inevitably lead to the reduction of CVDs risk. However, it should also be noted that the strength of the relationships between blood lipids/lipoproteins and CVD risk might be influenced by several lifestyle-related factors such as dietary patterns [28, 42] and socio-economic status [43]. For example, an increased daily intake of antioxidant and anti-inflammatory vitamins, polyunsaturated fatty acids and phytochemicals, either in the form of fruits, vegetables, fish and olive oil, or as nutraceuticals and supplements, is able to favorably modulate lipid levels and/or exert atheroprotective properties, irrespective of lipid status, by preventing the oxidizability of LDL [44, 45]. It is therefore, reasonable for someone to assume that the predictive ability of lipid indices will be lower in populations with a high consumption of the aforementioned dietary microconstituents. Although these factors were taken into account here (the MedDietScore reflects the daily intake of antioxidants and phytochemicals in the form of fruits and vegetables), cross-studies comparisons should be made with caution, because of the heterogeneity of the lifestyle and socio-economic status indices.

The importance of dyslipidemia is magnified by the fact that the independent associations of the aforementioned lipid indices with CVD risk were observed in a mildly dylipidemic cohort, as the current one, indicating the graded nature of the associations even around nearly normal values. One of the main goals of the present analysis was to reveal the best lipid biomarkers for accurate CVD risk prediction. Indentifying better predictors for a disease is of major importance for the correct disease management at individual, as well as at population level. However, it is interesting to see that numerous articles and book chapters on CVD epidemiology and prevention start with *“… cardiovascular disease is the main cause of death and disability, and … dyslipidemia is the trigger of atherosclerotic process that underlies CVD development*”; although this is true, the potential CVD candidate still remains under-estimated because of various reasons, the dyslipidemias are the most varied and there is a need to better diagnosing and treating dyslipidemia than prescribing lipid-lowering drugs [46]. Among all lipid indices measured in this study, the ratio of apoB/apoA1 demonstrated the best correct reclassification ability followed by non-HDL-C and TC/HDL-C ratio. Recent large prospective studies have shown that apoB is a better indicator of CVD risk than LDL-C [47, 48]. This is because apoB levels are a direct estimate of the atherogenic particles namely VLDL, IDL and LDL which all carry one molecule of apoB. The superiority of apoB against LDL-C as a CVD risk biomarker is more intense in populations which are characterized by insulin resistance and metabolic syndrome features. The apoB and LDL-C levels of those populations are discordant, due to the predominance of small dense LDL particles which contain less cholesterol than normal [49]. Therefore, the LDL-C levels of those populations may underestimate the real burden of the atherogenic particles. For similar reasons apoA1 is a better reflection of both HDL levels and functionality since the dense HDL3 subfraction along with pro-atherogenic HDL particles contain less apoA1 molecules [15]. In our study neither apoB nor apoA1 could not achieve a high cNRI, however their ratio (apoB/apoA1) was proved to have the best correct reclassification ability. The apoB/apoA1 ratio is actually an improved alternative to the TC/HDL-C ratio and its predictive ability for CVDs has been demonstrated before [50]. However, taking into account that: a) both TC/HDL-C and non-HDL-C show similar cNRIs with apoB/apoA1 ratio (Table 3), b) TC/HDL-C and non-HDL-C are calculated from standard lipid markers with no additional cost, c) the apoB and apoA1 measurements requires additional cost, d) the application of apoB and apoA1 measurements into routine clinical practise and prediction models requires a time-consuming and costly education of the clinicians, the incorporation of the apoB/apoA1 ratio into the daily practise is not justified, especially in countries under a financial crisis, like Greece.

### Strengths and limitations

The follow-up period of the study is quite long and the sample is representative of an urban Greek population. The panel of lipid indices, evaluated for their ability to predict CVD, was wide including both traditional and novel biomarkers. The models included a wide array of CVD risk determinants strengthening by this way the independent associations observed. Concerning the limitations of the study, the baseline evaluation was performed once, using questionnaires for lifestyle evaluation and, thus, may be prone to measurement errors. The sample of the study consisted of an urban population which cannot represent the total Greek population living in rural regions, too. The relatively small number of deaths and CVD events prevented from conducting sub-group analysis. Finally, no information on the use of dietary supplements and nutraceuticals was collected for the baseline examination of the ATTICA study, although, it is believed that, at that time, its use was not very popular, especially, on healthy individuals.

## Conclusion

In conclusion, this cohort study of CVD epidemiology in Greece demonstrated that elevated levels of lipid biomarkers are independently associated with all-cause mortality as well as CVD risk. These results suggest that, similarly to other North American and European populations, dyslipidemia is a strong independent risk factor for a Mediterranean population, too. The ratio of apoB/apoA1, followed by non-HDL-C, demonstrated the best correct classification ability of CVD risk, an observation that may be prone useful in better identifying the future CVD candidate at population level. Taking into account the difficulties and cost of a routine determination of both apoB and apoA1, the ability of non-HDL-C to improve risk stratification should be further evaluated. Since there is a large amount of data about the major causes of CVD, and there is unequivocal evidence that risk factor modification can reduce mortality, increase life expectancy and quality of life, there is a need to be able to correctly estimate CVD risk and plan effective, evidence based, public health strategies.
